# Occupational polycyclic aromatic hydrocarbons (PAHs) exposure is associated with accelerated aging trajectories in Chinese coke oven workers

**DOI:** 10.1038/s41598-026-36579-y

**Published:** 2026-01-31

**Authors:** Yidong Wang, Shuangxi Geng, Wenyu Wang, Lijun Yuan, Jisheng Nie, Huifang Zhang, Baolong Pan, Qiao Niu

**Affiliations:** 1 MOE Key Laboratory of Coal Environmental Pathogenicity and Prevention, NHC Key Laboratory of Pneumoconiosis, Department of Occupational Health, School of Public Health, Shanxi Key Laboratory of Environmental Health Impairment and Prevention, Shanxi Medical University, Xinjiannan Road 56, Taiyuan City, 030001 Shanxi Province China; 2 General Hospital of Taiyuan Iron & Steel (Group) Co., Ltd, Taiyuan, China

**Keywords:** Polycyclic aromatic hydrocarbons, Occupational exposure, Biological age, Aging trajectory, Prospective cohort study, Biomarkers, Environmental sciences, Health care, Medical research, Risk factors

## Abstract

Long-term effects of occupational polycyclic aromatic hydrocarbons (PAHs) on biological aging are unclear. A prospective cohort study was conducted from 2019 to 2023, involving 610 coke oven workers and 454 control workers from a water treatment plant. Biological age was calculated using the Klemera and Doubal method (KDM-BA) based on 12 clinical biomarkers, and aging acceleration (KDM-Accel) was derived. Group-based trajectory modeling (GBTM) was employed to identify distinct aging trajectories over five time points. The associations between eleven urinary mono-hydroxylated PAH metabolites (measured via HPLC-MS) and both KDM-Accel and aging trajectories were assessed using multiple linear regression and multivariable logistic regression, respectively. GBTM identified three distinct aging trajectories: slow aging (14.86%), moderately accelerated aging (59.14%), and rapidly accelerated aging (26%). Higher concentrations of urinary Σ-OHPAHs (sum of all metabolites), 1-hydroxypyrene (1-OHPyr), and 2-hydroxyphenanthrene (2-OHPhe) were significantly associated with increased KDM-Accel. A natural log-unit increase in Σ-OHPAHs and 1-OHPyr was associated with a 0.029-year and 0.028-year increase in KDM-Accel, respectively. Workers in the highest tertile (T3) of Σ-OHPAHs exposure had a 61.2% increased odds (OR = 1.612, 95% CI 1.093–2.376) of being in the rapidly accelerated aging trajectory compared to those in the lowest tertile (T1). Similar positive dose-response relationships were observed. Occupational exposure to PAHs, as specifically indicated by elevated levels of urinaryΣ-OHPAHs, 1-OHPyr, and 2-OHPhe, is correlated with accelerated biological aging and a heightened probability of a rapid aging trajectory. Therefore, enhanced protection measures and early intervention strategies are necessary.

**Supplementary Information:**

The online version contains supplementary material available at 10.1038/s41598-026-36579-y.

## Introduction

Polycyclic aromatic hydrocarbons (PAHs), a major component of air pollutants, originate from the incomplete combustion of organic matter. Composed of multiple fused benzene rings, PAHs form hydrocarbon substances with planar, angular, or cluster structures^1^.PAHs have been associated with a range of adverse health effects, including cardiovascular diseases^2^, cognitive dysfunction^3^, cancer^4^, metabolic abnormalities^5^, and increased mortality^6^. Furthermore, the consequences related to PAHs exposure are closely linked to the aging process.

Epidemiological studies indicate that PAHs exposure correlates with several biomarkers of aging.These include telomere shortening^7^, changes in DNA methylation patterns, increased mitochondrial DNA content^8^, and higher skin aging scores^9^. Such findings suggest that PAHs may play a role in accelerating both epigenetic and cellular aging processes^10,11^. However, it is important to note that the aforementioned aging-related biomarkers may have certain limitations.

In recent years, quantifying biological aging in conjunction with standard clinical parameters has been established as a highly accurate method for predicting diseases and mortality^12,13^. This approach is also becoming a key method for understanding how environmental exposures and interventions influence the risk of chronic diseases^14^. Unlike mere chronological aging, biological aging reflects the cumulative cellular and molecular damage that leads to functional decline^15^.

Recent analysis of the National Health and Nutrition Examination Survey (NHANES) database has revealed a correlation between exposure to mixed PAHs and increased aging^16^. Among these, 2-naphthol has been identified as a key component of PAHs associated with aging. Nonetheless, the precise relationship between PAHs exposure and biological age trajectories remains unclear. Specifically, it is not well understood how PAHs affect aging-related pathways in the early stages of exposure or to what extent they influence the aging process.

Additionally, when compared to the general population residing outside industrial areas, coal miners and coking plant workers are exposed to PAHs for longer durations and at higher intensities within more complex environmental mixtures. This chronic exposure may not only increase the risk of age-related diseases but also potentially accelerate biological aging itself, leading to the earlier onset of aging-related functional decline^3,17,18^.

Therefore, this study was designed as a prospective cohort study with the aim of assessing the impact of PAHs on biological age and aging trajectories. By developing a biological aging prediction model based on newly established biomarkers of biological aging and constructing aging trajectories, this study seeks to provide valuable insights into the long-term effects of PAHs exposure. The results of this research will offer scientific evidence for early disease intervention and risk stratification through the assessment of biological aging trajectories. Moreover, the findings will serve as an epidemiological basis to guide occupational workers in implementing effective protective measures to mitigate the health risks associated with PAHs exposure.

## Materials and methods

### Study participants

**Fig. 1:**
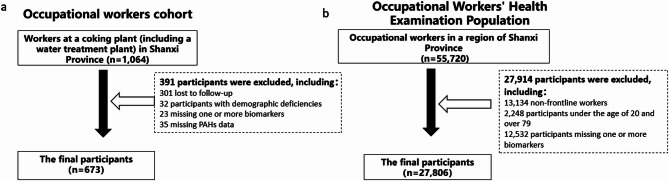
Flow chart of the study participant selection process.

This study used cluster sampling to build a occupational worker cohort, recruiting 610 coke oven workers and 454 from a water treatment plant in Shanxi Province in 2019. Follow-ups were done in 2020–2023. Participants were informed of the study’s purpose and procedures, advised to maintain a light diet, and required to fast for at least 12 h before sample collection. At enrollment, each participant completed a questionnaire on demographics, lifestyle, and medical history, conducted face-to-face by trained investigators using a standardized “Health Survey Form”.Inclusion criteria for study participants were as follows: (1) employment at the institution for at least one year; (2) general good health with no history of cancer, cardiovascular disease, stroke, or chronic syndromes such as multiple sclerosis, Alzheimer’s disease, Parkinson’s disease, depression, affective disorders, schizophrenia, or epilepsy, and no family history of neurological disorders; (3) willingness to provide written informed consent along with blood and urine samples. For aging trajectory analysis, 763 coking plant cohort participants aged ≥ 20 with baseline measurements in 2019 and four aging assessments (KDM-BA) were included.After excluding those with missing PAH exposure or demographic data, 673 subjects remained for longitudinal analysis to explor*e* the association between PAHs exposure and aging trajectories. The flowchart is shown in Fig. 1a.

We screened 55,720 occupational examinees in Shanxi, 2019. To enhance the representativeness of this cohort, non-frontline workers were excluded (*n* = 42,586). Second, to ensure that participants were of an age where age-related biomarker changes could be detected, while avoiding overrepresentation of groups with health statuses above or below the population average (e.g.those aged ≥ 80 years), the study population was restricted to individuals aged 20–79 years (*n* = 40,338). Third, participants with missing biomarker data were further excluded (*n* = 12,532), resulting in a final analytical sample of 27,806 individuals. The flowchart of the analytical sample selection is presented in Fig. 1b.

We use the occupational workers’ health examination population as the training dataset and the coking plant cohort as the test dataset to establish a biological aging predictor.This study was approved by the Medical Ethics Committee of the Shanxi Medical University and all participants signed informed consent form.The serial number is 2020GLL037.

### Baseline data collection

#### General questionnaire collection

The pre-enrollment questionnaire primarily covered seven sections: worker demographics (age, gender, ethnicity, height, weight, marital status, etc.), residential info, dietary habits (vegetables, fruits, meats, etc.), lifestyle habits (smoking, drinking, etc.), occupational history, medical history, and sleep patterns. Smokers are defined as those smoking ≥ 1 cigarette/day for ≥ 6 months; drinkers as ≥ 3 times/week for ≥ 1 year. Marital status includes single, married, etc. BMI is calculated as weight/height² (kg/m²). Each survey took ≤ 15 min to ensure quality.

#### Routine physical examination

All participants underwent a health examination, with blood samples and morning urine collected. Testing was done within 24 h, including biochemical tests, blood pressure, ECG, and ultrasound.

### Determination of urinary PAHs metabolites

During physical exams, professionals collected 50 mL urine samples using sterile cups. Samples were promptly transported to the lab, measured for specific gravity, and sub-packed into 5-mL cryotubes, then frozen at -80 °C. High-performance liquid chromatography-mass spectrometry (HPLC-MS) measured 11 PAH metabolites, including 2-OHNAP, 10-OHNAP, etc. Detailed procedures and quality control data are in previous studies(Table S1).Σ-OHPAHs is the sum of all 11 OH-PAH metabolites (urine-specific gravity-adjusted).

### Selection of biomarkers

Biomarker selection considered their role in aging, prior aging study applications, and availability in the occupational worker dataset, along with their Pearson correlation with chronological age. The 38 biomarkers analyzed fall into seven categories: metabolic function (BMI, blood sugar, etc.), cardiac function (blood pressure, heart rate), pulmonary function (FVC, FEV1, FEV1/FVC), renal function (urine pH, UA, etc.), liver function (ALT, AST), immune function (lymphocyte count, etc.), and blood cell counts (white blood cell count, etc.). Pearson correlation assessed the relationship between these biomarkers and age (Table S2).

Notably, platelet volume and count, hemoglobin, red blood cell count, hematocrit, systolic and diastolic blood pressure demonstrated high intercorrelation (*r* > 0.7). Following Klemera and Doubal’s dimensionality reduction approach, a single clinically relevant biomarker was systematically selected from each correlated parameter group as a representative indicator (e.g., platelet count for platelets, red blood cell count for erythrocytes, systolic blood pressure for blood pressure).Ultimately, 12 biomarkers were selected for biological age calculation, all with correlation coefficients > 0.1. These included ALT, RBC, HDL, TC, MCV, MCHC, RDW, PLT, LYC, BUR, and SBP.

### Statistical analysis

Data from questionnaires and standardized instruments were entered into a database using EpiData 3.1 software, with subsequent data cleaning performed in SPSS 25. Descriptive statistics were used to summarize the baseline characteristics of the study population during their initial physical examination. Socio-demographic characteristics were presented as mean ± standard deviation (X ± S) or frequency (percentage) (N%). Levels of polycyclic aromatic hydrocarbon hydroxy metabolites in urine were transformed using natural logarithm (Ln transformation) to achieve approximate normality for continuous variables, or categorized into three levels (T1, T2, T3) based on tertiles for categorical variables. Categorical variables were compared using chi-square tests, while continuous variables were analyzed with t-tests to assess differences in characteristics among different aging trajectory groups. Spearman correlation analysis was applied to evaluate the correlations among the 11 polycyclic aromatic hydrocarbons.

Biological age (KDM-BA) and biological aging acceleration (KDM-Accel) were precisely measured according to the method proposed by Klemera and Doubal. The prediction accuracy was assessed by calculating the Pearson correlation coefficient (R) and root mean square error (RMSE) between chronological age and KDM-BA. Moreover, to account for the effect of chronological age (CA), KDM-BA acceleration (KDM-Accel) was defined as the residual from the linear regression of KDM-BA on CA. The training dataset demonstrated a near-perfect Spearman’s rank correlation (ρ = 0.99) between chronological age and biological age in the occupational worker cohort, with RMSE = 0.205 years and MAE = 0.165 years (Fig. S1a). In the testing dataset focusing on the coking plant cohort, the correlation coefficient R2 was 0.99, with RMSE of 0.234 and MAE of 0.175,as seen in Fig. S1b. Both indicated good model predictability, verifying the effectiveness of the predictor. KDM-Accel is planned to be included as a biological aging indicator in subsequent analyses.

To explore the correlations between different hydroxy polycyclic aromatic hydrocarbon (OH-PAH) metabolites and the aging process, a multiple linear regression model was constructed, with the aging acceleration indicator (KDM-Accel) as the response variable and various OH-PAH metabolites (as continuous variables) as explanatory variables. The model adjusted for covariates including age, gender, education level, smoking status, alcohol consumption, tea consumption, exercise status, monthly income, shift work status, and other polycyclic aromatic hydrocarbon metabolites in urine. For missing covariate data, a dummy variable encoding method was used for missing categorical variables, and the median of continuous variables was used as a substitute for estimation. Additionally, restricted cubic spline models (RCS) were applied to analyze the dose-response relationship between polycyclic aromatic hydrocarbon exposure and the biological aging rate of workers.

KDM-Accel was calculated at five specific time points. Group-based trajectory modeling (GBTM) based on KDM-Accel was used to identify different aging trajectories at these five time points. The specific study design flow chart is shown in Fig.S2. The GBTM method is based on the assumption that the study population consists of different groups defined by individual developmental trajectories, and this process was implemented in SAS PROC TRAJ, with aging trajectories determined empirically. The number of subgroups of the biological aging index and the functional model for each group were determined by the following principles: (1) Bayesian Information Criterion (BIC), (2) average posterior probability of members (each biological aging trajectory class ≥ 0.7), and (3) reasonable distribution of study subjects in trajectory classes. A series of possible parameters for the GBTM model were considered, including fitting cubic, quadratic, and linear terms, with variations in one to six trajectory groups. The best-fitting trajectory model (3Group) was ultimately selected. These different aging trajectories consist of individuals with similar aging acceleration patterns, demonstrating the dynamic and heterogeneous process of aging. Specific parameter selection details are shown in Tables S3-S6 and Fig.S3.

This study further employs multiple logistic regression analysis to assess the association between the tertiles of exposure levels to polycyclic aromatic hydrocarbons (PAHs) and aging trajectories .The lowest tertile was used as the reference exposure level, and the slow-aging trajectory as the reference outcome category. Odds ratios (ORs) and their 95% confidence intervals (CIs) were calculated. Trend tests were performed by assigning the median of each tertile to the index and including it as a continuous variable in the model. Additionally, these indicators were included in the analysis as continuous variables (per 10-unit increase). To explore the impact of gender differences on the relationship between polycyclic aromatic hydrocarbons and aging trajectories, stratified analysis by gender was conducted. Multinomial logistic regression models were used to estimate ORs and 95% CIs, and further explored the relationships between the 11 polycyclic aromatic hydrocarbons and aging trajectories.

All analyses were conducted using SAS 9.7 (SAS Institute, Cary, NC), and figures were drawn using R 4.3.2. All p-values were two-sided. A p-value < 0.05 was considered statistically significant.The technology roadmap of this study is shown in Fig.S4.

## Results

### Basic characteristics

Among the 673 participants in the longitudinal study, the average age was 44.71 ± 7.32 years, and 89.45% were male(Table S7). Over half of the workers had an educational level above high school. Approximately 39.23% and 59.14% of the study subjects never smoked or drank alcohol, respectively, and 426 individuals (63.3% of the total) consumed tea. However, most subjects did not engage in healthy physical activities.

Compared with the control group, coking plant workers were younger and had a higher proportion of males (91.91% vs. 86.21%, *P* = 0.017), and a smaller proportion of those who often participated in physical exercise (32.64% vs. 41.03%, *P* < 0.001). No significant differences were found between the two groups in terms of educational level, marital status, smoking status, drinking status, tea drinking status, and night shift status (*P* > 0.05). Although there was no significant difference in KDM-Accel between the two groups, the average value was higher in the coking plant group than in the water treatment plant group.

The distribution of OH-PAHs in the urine of workers have been adjusted for urine specific gravity(Table S7). The detection rates of the 11 OH-PAHs metabolites were all above 75%. The geometric mean of OH-PAHs metabolites was calculated. The highest concentration of OH-PAHs metabolites was 2-OHNap (0.94 µg/L), and the lowest was 9-OHBap (0.07 µg/L). In the control group, the concentrations of OH-PAHs metabolites in the urine of water treatment plant workers were 9-OHPhe (0.37 µg/L) and 3-OHChr (0.04 µg/L). The levels of OH-PAHs in the urine of coking plant workers were consistently higher than those in the control group from the water treatment plant.

**Fig. 2:**
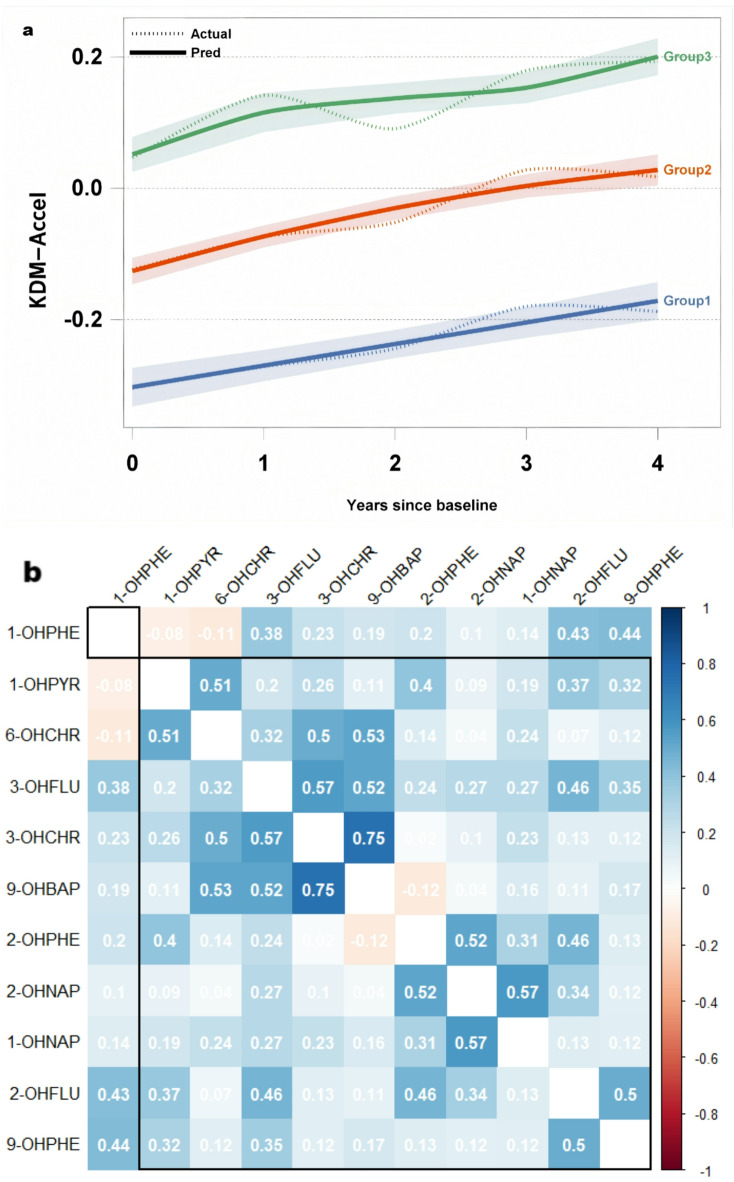

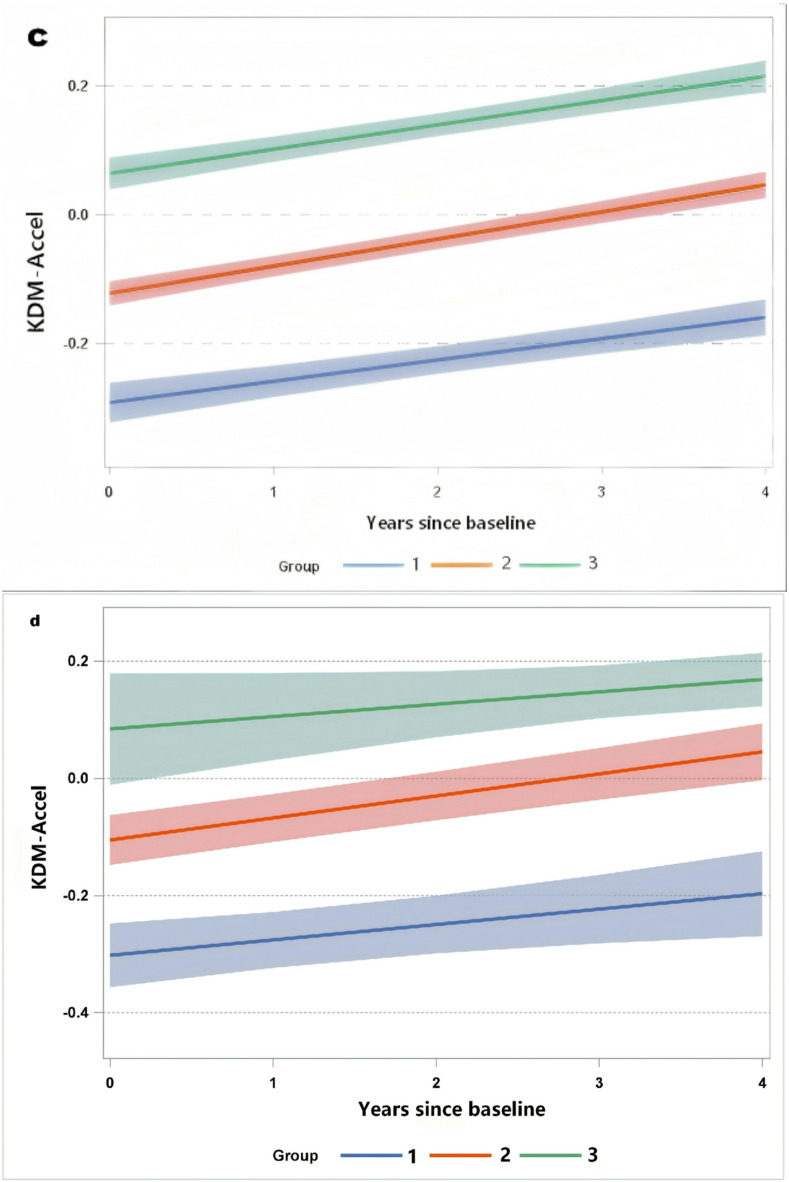
Biological aging trajectory chart and heatmap of correlation between PAHs metabolites in workers’ urine.

GBTM identified three potential categories of biological aging trajectories (Fig. 2a).Approximately 14.86% of the study subjects were classified into the slow aging trajectory group, all with negative KDM-Accel values, indicating that their biological age was generally younger than their chronological age across the five measures. About 59.14% were placed in the moderately accelerated aging trajectory group, showing moderate levels of accelerated aging initially. Individuals in this group had significantly higher urinary PAHs metabolite concentrations at baseline than those in the slow aging trajectory group. The remaining 26% were in the highly accelerated aging group, showing a high rate of accelerated aging across all four measures. The age of this group was 45.41 ± 7.12 years, and the concentration of PAHs metabolites in urine was the highest(Table 1). Figure 2b displays the pairwise Spearman’s rank correlations (*ρ*) among 11 polycyclic aromatic hydrocarbons (PAHs), with values ranging from 0.04 to 0.75. Specifically, 2-OHNap was moderately correlated with 2-OHPhe and 1-OHNap (*r* = 0.52, 0.57), and strongly correlated with 3-OHChr and 9-OHBap (*r* = 0.75). Sensitivity analysis was subsequently conducted, and aging trajectories were constructed separately for the coking plants and power plants using the GBTM method. The results showed that the trajectories of the two plants were homogeneous and showed increasing trends, with coking plants exhibiting a faster growth rate, a larger slope, and a higher KDM-Accel value than the control group (Fig. 2c and d).

1. Three trajectories have been identified among different populations: Slow aging(Group1), Moderate accelerated aging(Group2), and Highly accelerated aging(Group3). The different colored line segments represent different age trajectories. a.constructed for the total population, c .constructed for the population in the coking plant, d. constructed for the control group in the energy power plant.
2. The values in the b figure are Spearman correlation coefficients. The greater the absolute value of the correlation coefficient, the deeper the color.

**Table 1:** Basic characteristics of coke oven workers (*n* = 673).

variable	Total population *n* = 673	Aging trajectories			*P* value
		Slow aging group	Moderately accelerated aging group	Highly accelerated aging group	
		*n* = 100	*n* = 398	*n* = 175	
Age (years)	44.71 ± 7.32	44.61 ± 6.77	44.43 ± 7.53	45.41 ± 7.12	0.495
gender					**< 0.001**
man	602(89.45)	77(77.00)	357(89.70)	168(96.00)	
woman	71(10.55)	23(23.00)	41(10.30)	7(4.00)	
Educational attainment (years, N%)					**< 0.001**
1–9	176(26.15)	28(28.00)	100(25.13)	48(27.43)	
10–12	421(62.56)	61(61.00)	247(62.06)	113(64.57)	
≥ 13	76(11.29)	11(11.00)	51(12.81)	14(8.00)	
marital status (Yes, N%)					0.197
Yes	636(94.50)	93(93.00)	373(93.72)	170(97.14)	
No	37(5.50)	7(7.00)	25(6.28)	5(2.86)	
Smoking (Yes, N%)	409(60.77)	56(56.00)	254(63.82)	99(56.57)	0.150
Alcohol consumption (Yes, n%)	275(40.86)	47(47.00)	156(39.20)	72(41.14)	0.364
Tea drinking condition (Yes, n%)	426(63.30)	59(59.00)	254(63.82)	113(64.57)	0.618
Exercise status					0.676
never	186(27.64)	23(23.00)	115(28.89)	48(27.43)	
Occasionally	243(36.11)	38(38.00)	137(34.42)	68(38.86)	
often	244(36.26)	39(39.00)	146(36.68)	59(33.71)	
Night shift (yes, n%)	467(69.60)	70(70.00)	282(71.21)	115(65.71)	0.418
KDM (years)	44.61 ± 7.33	44.28 ± 6.77	44.30 ± 7.55	45.48 ± 7.12	0.127
kdm_advance (years old)	-0.11 ± 0.17	-0.33 ± 0.12	-0.13 ± 0.12	0.07 ± 0.11	**< 0.001**
Urine PAHs metabolites (µg/L)					
2-OHNap	0.68 ± 1.22	0.49 ± 0.77	0.67 ± 1.03	0.84 ± 1.71	0.544
1-OHNap	0.25 ± 1.16	0.17 ± 0.35	0.27 ± 1.39	0.27 ± 0.85	0.723
3-OHFlu	0.09 ± 0.15	0.07 ± 0.08	0.09 ± 0.13	0.11 ± 0.19	0.866
2 OHFlu	0.15 ± 0.18	0.13 ± 0.12	0.15 ± 0.16	0.16 ± 0.24	0.408
2-OHPhe	0.28 ± 0.47	0.19 ± 0.22	0.25 ± 0.35	0.39 ± 0.74	**0.046**
9-OHPhe	0.42 ± 0.55	0.35 ± 0.29	0.43 ± 0.50	0.45 ± 0.75	0.151
1-OHPhe	0.10 ± 0.16	0.09 ± 0.12	0.10 ± 0.13	0.11 ± 0.24	0.345
1-OHPyr	0.20 ± 0.25	0.14 ± 0.15	0.21 ± 0.26	0.23 ± 0.29	**0.049**
3-OHChr	0.06 ± 0.09	0.04 ± 0.04	0.05 ± 0.07	0.07 ± 0.13	0.627
6-OHChr	0.08 ± 0.08	0.07 ± 0.05	0.09 ± 0.07	0.09 ± 0.10	0.272
9-OHBap	0.06 ± 0.09	0.05 ± 0.05	0.06 ± 0.07	0.07 ± 0.12	0.473
Ʃ-OH PAHs	2.19 ± 2.67	1.65 ± 1.25	2.17 ± 2.46	2.57 ± 3.52	0.247

*P* < 0.05;

The continuous data were expressed as x ± s, and the statistical differences were compared by ANOVA.

The numerical data were expressed as n (%), and the chi-square test was used to compare the statistical differences.

### Association between occupational exposure and biological aging of workers exposed to polycyclic aromatic hydrocarbons

This study first explored the linear correlation between PAHs and the biological aging indicator (KDM-Accel). Using the natural logarithm-transformed PAH metabolites as continuous variables, we performed multiple linear regression analyses.Table 2 shows the results of the multiple linear regression model after controlling for covariables. The analysis indicates that exposure to 1-OHPyr and 2-OHPHE significantly increases workers’ biological age .Conversely, 2-OHFlu shows a negative correlation with biological age. Specifically, a one-unit natural log increase in 1-OHPYR concentration is associated with a 0.028-year increase in KDM-Accel; a similar increase in 2-OHPHE concentration corresponds to a 0.017-year rise. Conversely, a one-unit natural log increase in 2-OHNAP concentration is linked to a 0.026-year decrease in KDM-Accel. After further covariate adjustment, these effects weakened but remained statistically significant (*P* < 0.05). For total PAH metabolites, Model 1 adjusted for age, sex, and education. Results show that a one-unit natural log increase in Σ-OHPAHs is associated with a 0.029-year increase in KDM-Accel. In Model 2, further adjustment for smoking, drinking, tea consumption, exercise, monthly income, and night shift status shows a 0.032-year increase in KDM-Accel per one-unit natural log increase in Σ-OHPAHs.

**Table 2:** Relationship between urinary OH-PAHs metabolites and occupational workers’ biological aging index KDM-Accel.

In urine OH-PAHs metabolites	Multiple linear regression results β (95% CI)			
	Model one	*P*-value	Model two	*P*-value
Ʃ-OHPAHs ^a^	0.029(0.013,0.046)	**< 0.001**	0.032(0.015,0.049)	**< 0.001**
2-OHNAP ^b^	0.003(-0.007,0.013)	0.518	0.005(-0.005,0.015)	0.347
1-OHNAP ^b^	0.001(-0.013,0.015)	0.862	0.001(-0.013,0.015)	0.860
3-OHFLU ^b^	0.007(-0.005,0.018)	0.247	0.007(-0.005,0.018)	0.264
2-OHFLU ^b^	-0.026(-0.040,-0.011)	**< 0.001**	-0.022(-0.037,-0.007)	**0.004**
2-OHPHE ^b^	0.017(0.005,0.028)	**0.005**	0.014(0.002,0.026)	**0.019**
9-OHPHE ^b^	0.003(-0.0169,0.022)	0.736	0.005(-0.015,0.025)	0.608
1-OHPHE ^b^	-0.001(-0.014,0.011)	0.836	-0.003(-0.015,0.009)	0.615
1-OHPYR ^b^	0.028(0.011,0.045)	**0.001**	0.028(0.011,0.045)	**0.001**
3-OHChr ^b^	-0.012(-0.029,0.005)	0.175	-0.011(-0.028,0.006)	0.209
6-OHChr ^b^	-0.001(-0.014,0.013)	0.972	-0.001(-0.014,0.013)	0.900
9-OHBap ^b^	0.013(0.002,0.024)	**0.020**	0.012(0.001,0.023)	**0.042**

Model 1:adjusted for age, sex, and education level. b.adjusted for age, sex, education level, and other urinary OH-PAHs metabolites.

Model 2: adjusted for age, gender, education level, smoking status, drinking status, tea consumption status, exercise status, monthly income, and night shift status. b adjusted for age, gender, education level, smoking status, drinking status, tea consumption status, exercise status, monthly income, night shift status, and other urinary OH-PAHs metabolites. Bold indicates *P* < 0.05.

**Fig. 3:**
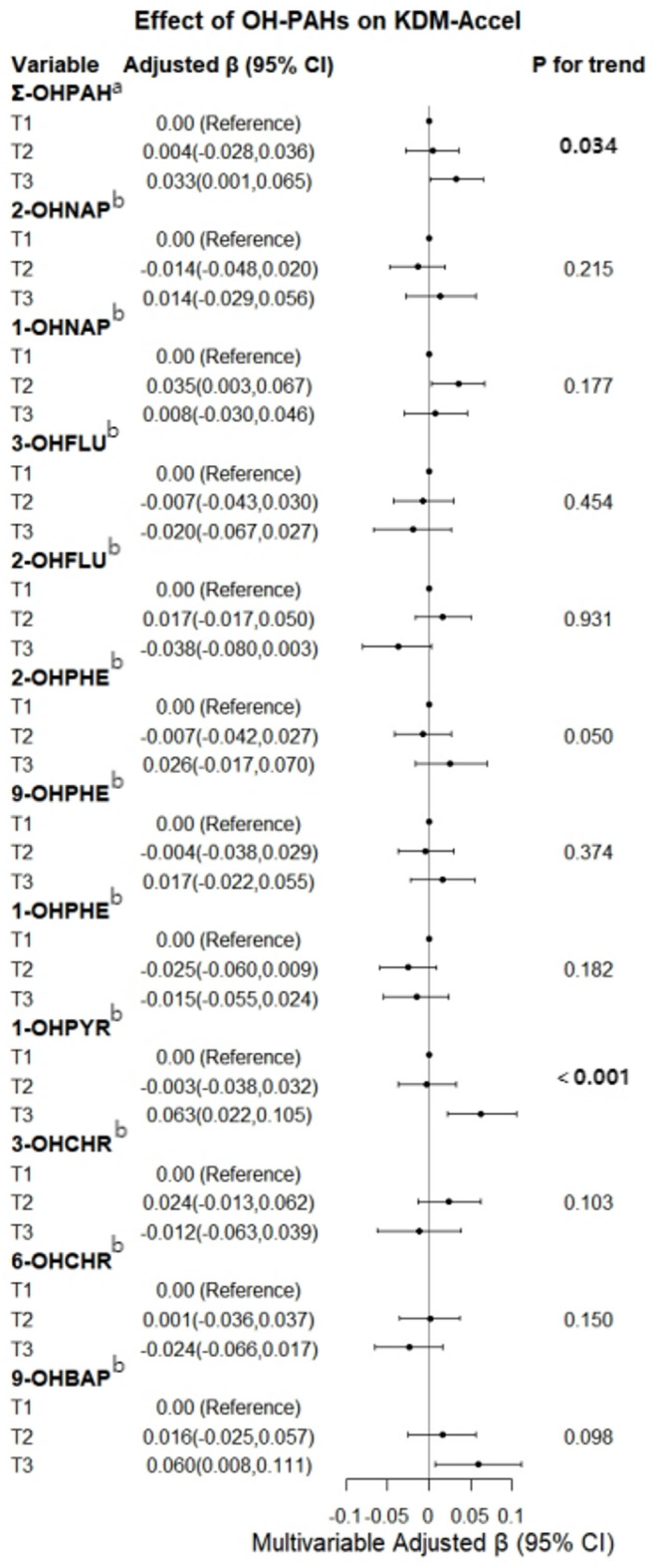
Relationship between OH-PAHs metabolites in urine and biological aging indicator KDM-Accel in occupational workers. Mode1: Adjust age, gender, education level, smoking status, alcohol consumption status, tea drinking status, exercise status, monthly income, night shift status. Mode2: Adjust age, gender, education level, smoking status, alcohol consumption status, tea drinking status, exercise status, monthly income, night shift status and other urinary OH-PAHs metabolites. Bold indicates *P* < 0.05.

Log-transformed concentrations of 11 individual PAH metabolites and Σ-OHPAHs were partitioned into tertiles based on their 33rd (P33) and 66th (P66) percentiles, defining three exposure strata: Group 1, Group 2,and Group 3.Categorical variables were included in the model for analysis. After adjusting for covariates, a generalized linear regression model was used to analyze the relationship between hydroxy-PAHs levels and KDM-Accel, as shown in Fig. 3. The model was adjusted for age, sex, education, smoking status, alcohol consumption, tea consumption, exercise status, monthly income, night shift status, and other urinary PAH metabolites. The analysis reveals that compared to Group 1, Group 3 of Σ-OHPAHs is associated with a 0.033-year increase in the workers’ biological aging indicator. Trend testing indicates a significant upward trend in the biological aging indicator with increasing urinary Σ-OHPAHs levels (*P* = 0.034). For 1-OHPyr, Group 3 versus Group 1 is associated with a 0.063-year increase in the biological aging indicator (OR = 0.063; 95%CI: 0.0215 ~ 0.1048). Trend testing also shows a significant upward trend in the biological aging indicator with increasing urinary 1-OHPyr levels (*P* = 0.006).

**Fig. 4:**
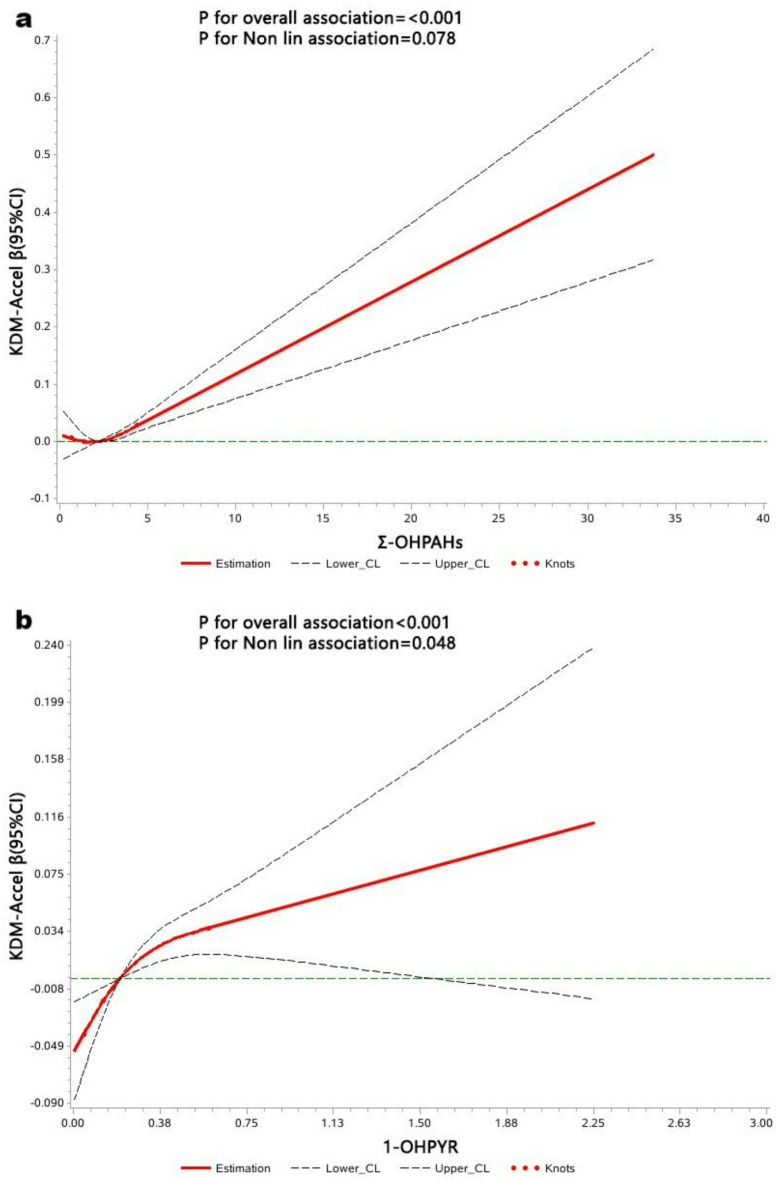
Dose-response relationship between urinary Ʃ-OHnap and biological aging indexes of workers in occupational workers. Adjusted covariates include age, gender, educational level, smoking status, alcohol consumption, physical exercise, frequency of night shifts, and other urinary OH-PAHs metabolites. The red line represents the dose-response relationship curve, with the dashed line indicating the 95% CI.

This study used restricted cubic splines to analyze the dose-response relationship between workers’ Σ-OHPAHs and the KDM-Accel. Using the median concentration of Σ-OHPAHs as the reference point, three knots (P_10_, P_50_, and P_90_) were selected for analysis, with results shown in Fig. 4a. A significant dose-response relationship was found between Σ-OHPAHs and the KDM-Accel (*P* < 0.001). As urinary Σ-OHPAHs concentrations increase, workers’ biological aging rates also increase in a linear dose-response manner. Additionally, using the median concentration of 1-OHPYR as the reference point and selecting three knots (P_10_, P_50_, and P_90_) for analysis, the results (Fig. 4b)indicate a significant dose-response relationship between 1-OHPYR levels and the KDM-Accel (*P* < 0.001). As urinary 1-OHPYR concentrations increase, workers’ biological aging rates also increase, exhibiting a non-linear dose-response relationship.

### Association between occupational exposure and biological aging trajectories in workers exposed to polycyclic aromatic hydrocarbons

#### Basic information about workers

The longitudinal data included 1064 workers who participated in the baseline survey and 673 workers who completed the follow-up. Table 3 compares the baseline information of 1064 workers with 673 workers included in the cohort study. The results showed that there were no statistically significant differences in age, sex, marital status, smoking status, drinking status, tea drinking status and night shift status between the two groups.

**Table 3:** Comparison of basic information between all workers at baseline (*n* = 1064) and workers included in the cohort (*n* = 673).

	Baseline: all workers (*n* = 1064)	Cohort inclusion study workers (*n* = 673)	*P* value
Age (years)	45.35 ± 7.09	44.71 ± 7.32	0.075
gender			0.647
man	959(90.13)	602(89.45)	
woman	105(9.87)	71(10.55)	
Marital status (yes, N%)			0.951
Tie the knot	1007(94.73)	638(94.80)	
Not married	56(5.27)	35(5.20)	
Smoking (Yes, N%)	671(63.06)	409(60.77)	0.337
Alcohol consumption (yes, n%)	447(42.05)	275(40.86)	0.624
Tea drinking status (yes, n%)	687(64.69)	425(63.15)	0.515
Night shift (yes, n%)	717(67.58)	467(69.60)	0.379

Bold represents *P* < 0.05;

The continuous data were expressed as x ± s, and the statistical differences were compared by ANOVA.

The numerical data were expressed as n (%), and the chi-square test was used to compare the statistical differences.

#### Association between occupational exposure and biological aging trajectories in workers exposed to polycyclic aromatic hydrocarbons

Table 4 shows the results of a multiple logistic regression model of the relationship between urinary OH-PAHs metabolites and accelerated aging trajectories with slow aging as the reference category. The results showed that the odds of being in the moderately accelerated aging increased by 39.2% for each increase of Ʃ-OHPAHs level in urine (OR = 1.392; 95%CI: 1.013 ~ 1.912), the odds of being in the highly accelerated aging trajectory increased by 61.2% (OR = 1.612; 95%CI: 1.093 ~ 2.376). For each additional natural logarithmic unit of 1-OHPyr level, the odds of being on a moderately accelerated aging trajectory increased by 47.2% (OR = 1.472; 95%CI: 1.058 ~ 2.048), the odds of being on a highly accelerated aging trajectory increased by 57.6% (OR = 1.576; 95%CI: 1.029 ~ 2.413). Similarly, for each additional natural logarithmic unit of 2-OHPhe levels, the odds of being on a highly accelerated aging trajectory increased by 46.7% (OR = 1.467; 95%CI: 1.075 ~ 2.001).Exposure to 2-OHFLU increasing by one natural logarithmic unit is associated with a lower likelihood of being in the moderately accelerated (OR = 0.602, 95% CI: 0.408–0.887) or highly accelerated trajectory(OR = 0.543, 95% CI: 0.334–0.882) .

**Table 4:** Relationship between urinary OH-PAHs metabolites and aging trajectories of occupational workers.

Urine metabolites of OH-PAHs	Moderate aging vs. slow aging		High aging vs. slow aging	
	OR (95% CI)	*P*	OR (95% CI)	*P*
Ʃ-OHPAHs ^a^	**1.392(1.013**,**1.912)**	**0.041**	**1.612(1.093**,**2.376)**	**0.016**
2-OHNAP ^b^	1.112(0.903,1.370)	0.316	1.011(0.801,1.276)	0.927
1-OHNAP ^b^	0.901(0.672,1.209)	0.487	0.957(0.692,1.323)	0.790
3-OHFLU ^b^	1.207(0.955,1.526)	0.115	1.211(0.926,1.583)	0.162
2-OHFLU ^b^	**0.602(0.408**,**0.887)**	**0.010**	**0.543(0.334**,**0.882)**	**0.014**
2-OHPHE ^b^	1.098(0.882,1.366)	0.405	**1.467(1.075**,**2.001)**	**0.016**
9-OHPHE ^b^	1.102(0.743,1.634)	0.628	1.195(0.757,1.887)	0.445
1-OHPHE ^b^	1.109(0.864,1.425)	0.417	0.856(0.620,1.180)	0.342
1-OHPYR ^b^	**1.472(1.058**,**2.048)**	**0.022**	**1.576(1.029**,**2.413)**	**0.036**
3-OHChr ^b^	0.733(0.536,1.003 )	0.052	0.779(0.520,1.166)	0.225
6-OHChr ^b^	1.139(0.894,1.451)	0.291	0.964(0.696,1.335)	0.825
9-OHBap ^b^	1.060(0.859,1.308)	0.586	1.263(0.945,1.688)	0.114

*OR*: odds ratio; CI: Confidence interval. Slow Aging: Slow Aging Trajectory, Moderate Aging: Moderately Accelerated Aging Trajectory, High Aging: Highly Accelerated Aging Trajectory. Model: aAdjust age, gender, education level, smoking status, alcohol consumption status, tea drinking status, exercise status, monthly income, night shift status. bAdjust for age, sex, education, smoking, drinking, tea, exercise, monthly income, night shift, and other urinary OH-PAHs metabolites. Bold indicates *P* < 0.05.

The 11 ln-transformed OH-PAHs metabolites were categorized into three groups based on their respective P_33_ and P_66_ percentiles, with biological aging trajectory modeled as a categorical variable. The lowest tertile served as the exposure reference, and slow aging as the outcome reference. Multiple logistic regression analyses demonstrated that subjects in the highest tertile of Σ-OHPAHs and 1-OHPyr were respectively 83.4% (OR = 1.834, 95% CI: 1.024–3.283, *P* = 0.034) and 88.6% (OR = 1.886, 95% CI: 1.033–3.444, *P* = 0.037) more likely to be on a moderately accelerated aging trajectory compared to the lowest one-third. Those with the highest third of 3-OHChr had a 64.2% decreased risk of being on a moderately accelerated aging trajectory (OR = 0.358, 95% CI: 0.143–0.897), yet the trend test was insignificant. For highly accelerated aging trajectories, subjects in the highest one-third of Σ-OHPAHs, 2-OHPhe, and 1-OHPyr had respective odds of 2.002 (OR = 2.002, 95% CI: 1.008–3.978, *P* = 0.04), 3.374 (OR = 3.374, 95% CI: 1.133–10.052, *P* = 0.043), and 2.849 (OR = 2.849, 95% CI: 1.003–8.095, *P* = 0.043). These results are detailed in Table 5.

**Table 5:** Relationship between urinary OH-PAHs metabolites and aging trajectories of occupational workers.

Urine metabolites of OH-PAHs	Moderate aging vs. slow aging		High aging vs. slow aging	
	OR (95% CI)	P_trend	OR (95% CI)	P_trend
Ʃ-OHPAHs^a^				
T1	1.000(Reference)		1.000(Reference)	
T2	1.047(0.615,1.783)	**0.034**	1.239(0.634,2.420)	**0.043**
T3	**1.834(1.024**,**3.283)**		**2.002(1.008**,**3.978)**	
2-OHNAP^b^				
T1	1.000(Reference)		1.000(Reference)	
T2	0.570(0.299,1.087)	0.787	**0.397(0.163**,**0.967)**	0.439
T3	0.834(0.354,1.964)		0.744(0.244,2.263)	
1-OHNAP^b^				
T1	1.000(Reference)		1.000(Reference)	
T2	1.815(0.977,3.371)	0.223	**3.217(1.280**,**8.086)**	0.301
T3	1.695(0.818,3.514)		1.887(0.698,5.102)	
3-OHFLU^b^				
T1	1.000(Reference)		1.000(Reference)	
T2	1.100(0.551,2.195)	0.873	1.184(0.475,2.950)	0.976
T3	0.707(0.292,1.711)		0.988(0.301,3.243)	
2-OHFLU^b^				
T1	1.000(Reference)		1.000(Reference)	
T2	0.782(0.414,1.478)	0.712	0.660(0.274,1.590)	0.761
T3	0.688(0.292,1.711)		0.367(0.124,1.088)	
2-OHPHE^b^				
T1	1.000(Reference)		1.000(Reference)	
T2	0.983(0.521,1.857)	0.101	1.274(0.546,2.972)	**0.034**
T3	2.000(0.815,4.908)		**3.374(1.133**,**10.052)**	
9-OHPHE^b^				
T1	1.000(Reference)		1.000(Reference)	
T2	1.478(0.772,2.832)	0.296	0.488(0.203,1.174)	0.830
T3	1.385(0.652,2.942)		0.912(0.342,2.433)	
1-OHPHE ^b^				
T1	1.000(Reference)		1.000(Reference)	
T2	0.818(0.414,1.615)	0.711	0.686(0.273,1.727)	0.075
T3	1.018(0.456,2.271)		0.611(0.218,1.717)	
1-OHPYR^b^				
T1	1.000(Reference)		1.000(Reference)	
T2	0.973(0.567,1.668 )	**0.037**	0.719(0.302,1.713)	**0.019**
T3	**1.886(1.033**,**3.444 )**		**2.849(1.003**,**8.095)**	
3-OHChr^b^				
T1	1.000(Reference)		1.000(Reference)	
T2	0.502(0.239,1.052 )	0.289	0.935(0.360,2.428)	0.992
T3	**0.358(0.143**,**0.897 )**		0.245(0.057,1.048)	
6-OHChr^b^				
T1	1.000(Reference)		1.000(Reference)	
T2	1.633(0.819,3.256)	0.196	1.528(0.583,4.006)	0.289
T3	0.901(0.405,2.003)		0.617(0.202,1.882)	
9-OHBap^b^				
T1	1.000(Reference)		1.000(Reference)	
T2	2.249(0.981,5.156)	0.393	2.798(0.961,8.146)	0.166
T3	**3.23(1.259**,**8.283)**		**9.034(1.909**,**42.753)**	

OR: odds ratio; CI: Confidence interval. Slow Aging: Slow Aging Trajectory, Moderate Aging: Moderately Accelerated Aging Trajectory, High Aging: Highly Accelerated Aging Trajectory. Model: aAdjust age, gender, education level, smoking status, alcohol consumption status, tea drinking status, exercise status, monthly income, night shift status. bAdjust for age, sex, education, smoking, drinking, tea, exercise, monthly income, night shift, and other urinary OH-PAHs metabolites. Bold indicates *P* < 0.05.

## Discussion

This study undertook the development of a biological age predictor for Chinese workers and assessed the correlation between Polycyclic aromatic hydrocarbons (PAHs) exposure and aging biomarkers.In a prospective cohort study, the research successfully applied group-based trajectory modeling (GBTM) techniques to identify three distinct aging trajectories within the study sample, thereby evaluating the rate of aging. Biological age(BA), as a more precise measure of aging than chronological age, integrates various biomarkers related to physical function and provides comprehensive information about overall health. The results indicate that individuals exposed to high levels of PAHs tended to be biologically older than those exposed to low levels. Specifically, each natural logarithmic unit increase in Σ-OHPAHs raised the likelihood of moderate and high accelerated aging trajectories by 39.2% and 61.2%, respectively.

Human aging likely begins at or before reproductive maturity Identifying modifiable risk factors is crucial for maintaining health and quality of life during aging^19^. Chronological Age (CA) serves as a convenient method for assessing the aging status of individuals, but it cannot accurately reflect the differences in aging rates among individuals within the same CA group. The variation in cognitive function and health status increases with the growth of CA, indicating that CA may not accurately reveal the true aging differences between individuals.The proposed biological age (BA) describes aging relative to CA^20^.

The estimation of biological age (BA) can be facilitated by omics indicators (such as telomere length)^21^, clinical indicators measurement constructed based on the Klemera and Doubal method)^22^, and phenotypic indicators (such as frail phenotype, Frailty Index (FI), and functional aging index)^23–25^.This study employed a BA method constructed based on clinical indicators, which has the advantage of using CA as a standard biomarker in the model, significantly enhancing the accuracy of BA estimation, and elucidating the known “biomarker paradox” correlation. The study demonstrated that incorporating CA as a biomarker into the model significantly reduced the standard error of BA estimation. In predicting mortality and disease, the KDM method has been proven superior to other traditional methods^26^. In 2020, a longitudinal cohort study of Singaporean Chinese compared the differences in BA prediction of aging, disease, and mortality using MLR, PCA, and KDM methods^27^. The results showed that the KDM method’s BA had significant predictive power in the younger group, and for groups with the same CA, the group with accelerated aging had a higher incidence of disease, worse health conditions, and more severe biological functional aging than the group with slower aging. This study applied the KDM method to construct BA in the population of Chinese occupational workers, and the results showed that KDM-BA highly matched with CA. In our study, the constructed KDM-BA included biochemical markers of liver function, kidney function, cardiovascular function, metabolic function, lipids, inflammation, and metabolism, which allows us to more comprehensively identify changes in the workers’ bodies and distinguish earlier those in a preclinical state.Studies have shown that even at baseline without ischemic heart disease, stroke, or cancer, KDM-Accel to some extent distinguished individuals who may be in the subclinical stage of cardiovascular disease (CVD), indicating that KDM-Accel can reflect preclinical content earlier and better reflect early changes in the human body.

Environmental factors, including air pollution, have a potential impact on the biological aging process. Long-term exposure to air pollution is considered one of the key factors leading to adverse age-related outcomes, particularly cardiovascular diseases (CVD) and respiratory system diseases^28^.Studies have shown that exposure to air pollution may even shorten the expected lifespan of individuals with good genetic endowment. Polycyclic aromatic hydrocarbons (PAHs), comprising hundreds of chemically related compounds with diverse structures and toxicities, including carcinogenicity and neurotoxicity, persistently pollute the environment. Exposure in occupational environments may involve multiple pathways, affecting the total absorbed dose (e.g.skin and inhalation exposure to contaminated air)^29,30^. People can be exposed to PAHs through direct inhalation, ingestion, or skin contact with air and surface soils containing these compounds^31^. Workers in coking plants are commonly exposed to PAHs. This study selected the content of PAH metabolites in urine as an indicator of human exposure, primarily based on its high specificity as a biomarker compared to blood samples, as well as the non-invasive collection advantages of urine samples.Given the complexity of PAHs in coking plants, 11 PAH hydroxy metabolites were used as a comprehensive exposure index.

PAHs cause mitochondrial dysfunction, oxidative stress^32^, and DNA damage^33^, thereby leading to cellular aging and premature bodily aging^34^. Oxidative stress and the resulting chronic inflammation have been confirmed as significant pathogenic factors for the aging process and age-related diseases^17^.Previous studies have demonstrated that PAHs exposure induces oxidative stress, which refers to an imbalance between the production of oxidants and antioxidant defenses in the body^35^. This oxidative stress ultimately leads to aging and disease.PAHs bind to AhR receptors, inducing P450 enzymes to generate a large quantity of reactive oxygen species (ROS) during cellular metabolism^36^.These ROS induce changes in antioxidant enzymes (GST, GPx, GR, and total glutathione)^37^.Through these alterations in glutathione biosynthesis, the antioxidant defense system is modified, resulting in increased ROS production, oxidative stress, and ultimately accelerated aging. Some cell line models have also demonstrated the same outcome, where the neurotoxicity of B[a]P is attributed to severe oxidative stress^38^.During cellular metabolism, B[a]P undergoes intracellular biotransformation into active intermediates through the activation of diol epoxides by cytochrome P450 (CYP) enzymes, thus generating reactive oxygen species (ROS).These studies suggest that B[a]P may influence neuronal behavior in adult rats by inducing cellular oxidative stress and disrupting the balance between ROS and antioxidant production. This disruption ultimately accelerates aging in the animals.Epidemiological studies have shown that exposure to PAHs is associated with oxidative stress, even in low-exposure environments^39^.

Our study for the first time reveals the correlation between high exposure to PAHs and accelerated biological aging. Specifically, for each additional natural logarithmic unit of Ʃ-OHPAHs, the biological aging indicator of workers increases by 0.029 years; for each additional natural logarithmic unit of 1-OHPYR concentration, the biological aging indicator KDM-Accel increases by 0.028 years; for each additional natural logarithmic unit of 2-OHPHE concentration, the biological aging indicator KDM-Accel increases by 0.017 years. The results of this study are consistent with previous studies that workers occupationally exposed to PAHs exhibit shorter telomere lengths and accelerated lymphocyte DNA methylation age compared to those exposed to less PAHs^11^; long-term skin exposure to PAHs may lead to higher skin aging scores^40^; 1-OHPYR and 9-OHPHE are associated with methylation aging independently of other OH-PAHs and risk factors^41^.

Furthermore, our study represents a significant advancement compared to previous research. First, we used objectively measured urinary OH-PAH metabolites to quantify the total exposure to PAHs from various sources, including diet, cooking, smoking, heating, transportation, waste burning, and occupational exposure. The use of OH-PAH metabolites not only improves the accuracy of exposure measurement but also allows us to distinguish the effects of different types of PAHs. Furthermore, among our study participants, we did not observe a significant correlation between smoking and biological aging; this observation may be partly due to the fact that smoking largely leads to exposure to naphthalene rather than phenanthrene and pyrene^42^. Second, compared to previous cellular aging indicators, biological age provides additional information—it has a similar scale to actual age and can be linked to the epigenetic behavior of certain age-related genes^43^. After multiple logistic analyses by gender grouping, the results showed that the male group was consistent with the total population(Table S8). However, in females, only 2-OHFLU showed consistent results, while Ʃ-OHPAHs were not statistically significant for the aging trajectory(Table S9). One possible reason is that estrogen has antioxidant and anti-inflammatory effects, and the biological aging caused by PAH exposure is through oxidative stress and chronic inflammation^44^, which leads to a smaller impact of PAHs on the biological aging indicators of females^45^. Another important factor is that after stratification by gender, there were fewer female samples, and therefore, the impact of PAHs on the biological aging indicators of the female group could not be observed.

Regarding the trajectory of aging, early studies identified different patterns of human aging trajectories by measuring indices such as frailty index^46^, health and functional items^47,48^, physical capacity, and educational items^49^, or other subjective indicators^50^. Compared with previous studies, our current research demonstrates the feasibility of long-term estimation of aging speed in the population of Chinese occupational workers. Compared to measuring aging at a single time point, the biological aging trajectory established based on aging biomarkers measured over a longer period may better capture the dynamics and heterogeneous nature of aging and is expected to accurately identify different populations with varying risks of age-related diseases or mortality. In the study, it was found that as the time of enrollment increased, the trajectory curve gradually rose. A possible explanation is that our population consists of workers under the age of 60, and most people experience a certain degree of chronic diseases around the age of 60^51^, with exposure to PAHs increasing the risk of such diseases. This will lead to an increasing trend in biological age over time for our study subjects. Previous studies constructed biological aging trajectories based on functional decline indicators, and the results showed that with the increase of time, the aging trajectory presented an upward trend, which is consistent with our study. We reconstructed the biological aging trajectory charts for coking plant workers and the control group of water treatment plant workers, and consistent results were found, both showing an increasing trend. Moreover, the aging trajectory chart constructed for coking plant workers showed a higher increase trend and a steeper slope than the aging trajectory of the control group water treatment plant, indicating that the biological aging trajectory may be caused by exposure to PAHs, resulting in a higher increase trend. Our results show that for every natural logarithmic unit increase in the urinary level of Σ-OHPAHs, the likelihood of being in a moderately accelerated aging trajectory increases by 39.2% (OR = 1.392; 95%CI: 1.013 ~ 1.912), and the likelihood of being in a highly accelerated aging trajectory increases by 61.2% (OR = 1.612; 95%CI: 1.093 ~ 2.376). For every natural logarithmic unit increase in 1-OHPyr, the likelihood of being in a moderately accelerated aging trajectory increases by 47.2% (OR = 1.472; 95%CI: 1.058 ~ 2.048), and the likelihood of being in a highly accelerated aging trajectory increases by 57.6% (OR = 1.576; 95%CI: 1.029 ~ 2.413). Additionally, for every natural logarithmic unit increase in 2-OHPHE, the likelihood of being in a highly accelerated aging trajectory increases by 46.7% (OR = 1.467; 95%CI: 1.075 ~ 2.001). We have further confirmed from the perspective of trajectories that PAHs can accelerate the process of biological aging.Accelerated aging is a multi-system aging process with complex underlying biological mechanisms. Our findings may provide a new approach to studying how environmental pollution induces adverse health outcomes related to aging (including age-related diseases and mortality) through aging.This has public health significance for preventing diseases in occupational workers.

Our study has several advantages. Firstly, we conducted a prospective cohort study. Secondly, we conceptualized biological aging acceleration using multiple indicators, which are based on 12 biomarkers from routine examinations representing multiple organ systems. Thirdly, compared to a single time point assessment, we tracked aging acceleration at five time points using Group-Based Trajectory Modeling (GBTM), which may be more suitable for evaluating the aging process. The GBTM model explains the changes over time, differentiates the acceleration of aging over time, and is capable of identifying the heterogeneity of aging.

## Study limitations

However, this study has certain limitations. Firstly, we only conducted a single measurement of polycyclic aromatic hydrocarbons (PAHs) in urine, but since urinary PAH metabolites can reflect long-term internal exposure indicators, PAHs have been used as internal exposure indicators in previous studies. Secondly, we tried to control all confounding factors, but due to the observational nature of the study, the possibility of residual confounding cannot be ruled out. Thirdly, for certain specific metabolites (e.g., 9-OHBap), the observed odds ratios showed wider confidence intervals, which may reflect limited statistical precision in exposure quantile or subgroup analyses. These findings should be interpreted cautiously and validated in larger-scale studies.Fourthly, because our correlation analysis was conducted in a cohort of occupational workers, biological aging was only measured in whole blood, so our correlation results may not be generalizable to other non-worker populations or to biological aging in other tissues. Fifthly, our cohort study had a relatively short duration, allowing us to observe only the short-term relationship between PAHs and biological aging trajectories. Finally, our study population had a relatively small proportion of females. We plan to increase the number of female subjects in future studies to observe the impact of PAHs on biological aging in females.

## Conclusion

In summary, our study provides evidence that workers with higher exposure to polycyclic aromatic hydrocarbons (PAHs) experience accelerated aging compared to those with lower exposure. PAHs levels are positively associated with biological aging indicators and trajectories. Occupational PAHs exposure may predispose workers to accelerated aging. Notably, exposure to 1-OHPyr, 2-OHPhe, and Σ-OHPAHs was significantly associated with accelerated biological aging, indicating that these specific PAH metabolites may be key drivers of occupational aging.Reducing PAHs exposure could potentially reduce the aging rate among coke oven workers. Future longitudinal studies should employ larger samples with broader demographic representation to validate these findings and develop targeted interventions against occupationally accelerated aging.

## Supplementary Information

Below is the link to the electronic supplementary material.

Supplementary Material 1

## Data Availability

The data that support the findings of this study are not publicly available due to their containing information that could compromise the privacy of research participants. The data are available from the corresponding author upon reasonable request.
