# Supplementary material for: Occupational polycyclic aromatic hydrocarbons (PAHs) exposure is associated with accelerated aging trajectories in Chinese coke oven workers
Source: Sci Rep. 2026 Jan 31;16:6852. doi: 10.1038/s41598-026-36579-y (PMC12916824; doi:10.1038/s41598-026-36579-y)
Supplement: Supplementary file 1 — Supplementary Material 1 [file 41598_2026_36579_MOESM1_ESM.docx]

| PAHs metabolites | ROC（R²） | Detection limit（LOD,μg/L） | Detection rate（%） | Spiked recovery rate（%） | RSD |
| --- | --- | --- | --- | --- | --- |
| 2-0HNap | 0.9993-1.0000 | 0.0053 | 96.8% | 101.00-127.13 | 11.05-18.78 |
| 1-0HNap | 0.9991-0.9999 | 0.024 | 93.9 | 100.22-123.67 | 3.84-7.85 |
| 3-OHFlu | 0.9990-1.0000 | 0.0041 | 93.9 | 97.59-126.83 | 3.06-3.73 |
| 2-OHFlu | 0.9992-0.9999 | 0.0017 | 97.9 | 95.08-129.48 | 3.47-4.84 |
| 2-0HPhe | 0.9991-1.0000 | 0.0014 | 98.3 | 92.44-137.99 | 2.83-3.28 |
| 9-0HPhe | 0.9991-1.0000 | 0.0441 | 98.7 | 93.02-114.149 | 3.75-10.16 |
| 1-OHPhe | 0.9990-1.0000 | 0.0142 | 78.9 | 88.31-128.86 | 5.58-6.52 |
| 1-0HPyr | 0.9990-0.9999 | 0.0098 | 97.6 | 122.81-125.35 | 6.26-9.87 |
| 3-0HChr | 0.9992-1.0000 | 0.0023 | 94.9 | 111.28-126.5 | 6.97-7.74 |
| 6-OHChr | 0.9989-1.0000 | 0.0041 | 88.9 | 110.84-111.83 | 6.80-9.90 |
| 9-OHBap | 0.9990-1.0000 | 0.0027 | 76.5 | 119.13-134.57 | 5.56-5.88 |

**Table S1 Laboratory quality control data for metabolites of PAHs in urine**

| Biomarker | Pearson correlation coefficient between chronological age (CA) and biomarkers |
| --- | --- |
| BMI | -0.022^**^ |
| Heart rate | 0.074^**^ |
| FVC | 0.047^**^ |
| FEV1 | -0.077^**^ |
| FEV1/FVC | 0.077^**^ |
| Systolic blood pressure（SBP） | 0.312^**^ |
| Diastolic blood pressure（DBP） | 0.103^**^ |
| Alanine transferase（ALT） | -0.123^**^ |
| Aspartate aminotransferase（AST） | 0.018^**^ |
| Total cholesterol（TC） | 0.132^**^ |
| Triglyceride（TG） | 0.023^**^ |
| High density lipoprotein（HDL） | 0.114^**^ |
| Low density lipoprotein（LDL） | 0.093^**^ |
| White blood cell count（WBC） | -0.067^**^ |
| Urine pH（PH） | 0.056^**^ |
| Erythrocyte（RBC） | -0.257^**^ |
| Hemoglobin（HGB） | -0.192^**^ |
| Hematocrit（PCT） | -0.208^**^ |
| Hematocrit（HCT） | -0.146^**^ |
| Mean corpuscular volume（MCV） | 0.215^**^ |
| Mean corpuscular hemoglobin（MCH） | 0.074^**^ |
| Mean corpuscular hemoglobin concentration（MCHC） | -0.197^**^ |
| Red blood cell distribution width （RDW） | 0.352^**^ |
| Coefficient of variation of red blood cell distribution width（RDW-CV） | 0.074^**^ |
| Platelet count（PLT） | -0.170^**^ |
| Platelet distribution width（PDW） | 0.024^**^ |
| Mean platelet volume（MPV） | 0.030^**^ |
| Large platelet ratio（PLCR） | 0.091^**^ |
| Lymphocyte count（LYC） | -0.148^**^ |
| Neutrophil count（NEUT） | -0.030^**^ |
| Eosinophil number（EO） | 0.023 |
| Basophil number（BASO） | -0.038^**^ |
| White blood cell count（MXD） | 0.033^**^ |
| Monocyte count（MONO） | 0.016 |
| Uric acid（UA） | -0.086^**^ |
| Serum creatinine（SCR） | 0.038^**^ |
| Urea nitrogen（BUR） | 0.136^**^ |
| Blood glucose（GLU） | 0.253^**^ |
| 注: ***p <0 .0001. **p <0 .01. *p < 0.05. |  |

**Table S2 Pearson correlation coefficient between chronological age (CA) and biomarkers**


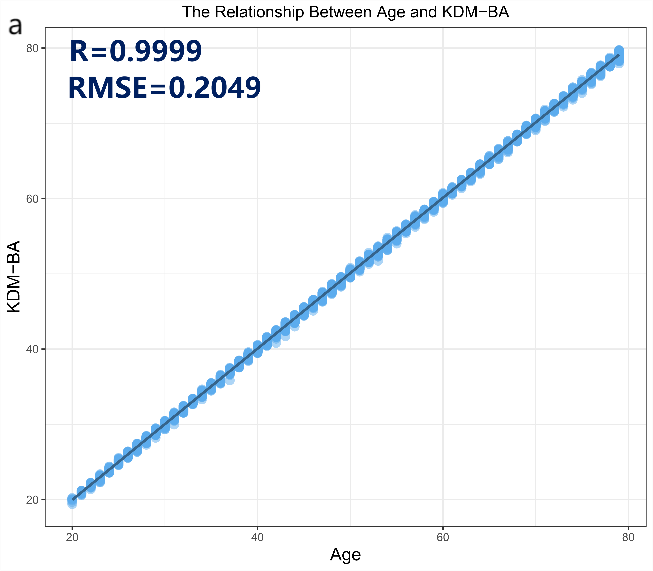

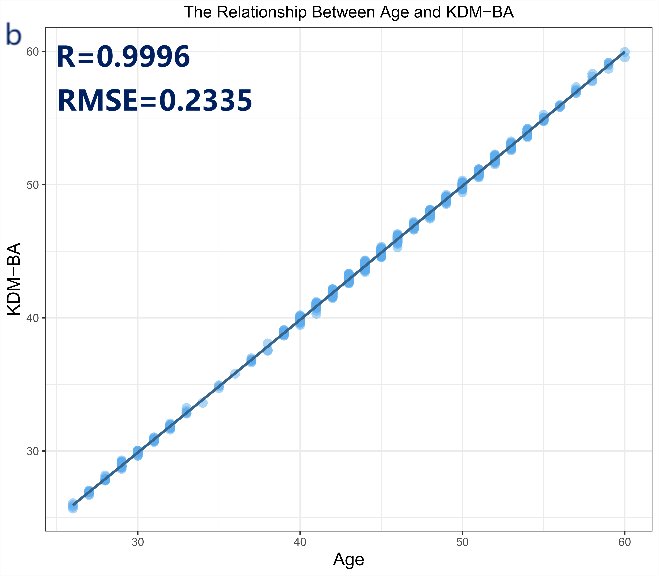


**Fig.S1.The relationship between chronological age and KDM-BA**

(a) Training dataset; (b) Test dataset.


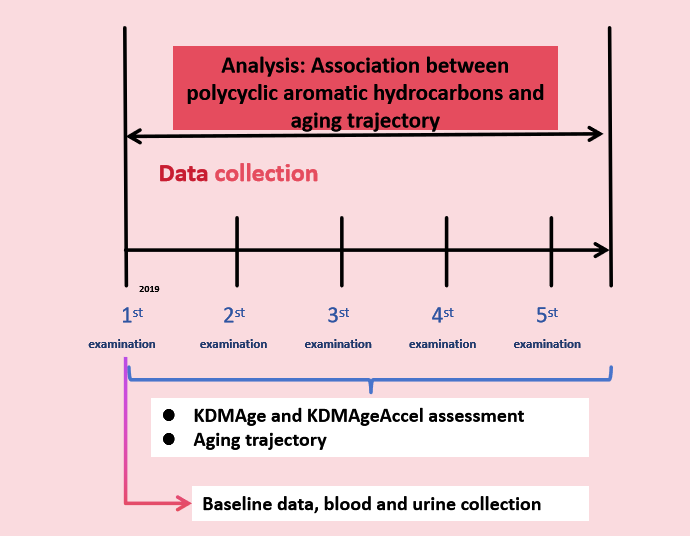


**Fig.S2. Diagram of coking plant cohort study data collection time**

| Group | *Avepp*（%） | *OCC* | *P_j_*（%） | *π_j_*（%） | *BIC^#2^* | *△BIC^#2^* | *E_k_* |
| --- | --- | --- | --- | --- | --- | --- | --- |
| 1Group （2 ） | 100.00 |  | 100.00 | 100.00 | 749.29 |  | 0.000 |
| 2Group （1 1 ） | 87.24-89.53 | 9.5-6.2 | 41.01-58.99 | 41.95-58.05 | 992.92 | 243.63 | 0.636 |
| 3Group （1 2 3 ） | 85.72-84.17-83.78 | 30.2-4.2-13.7 | 14.86-59.14-26.00 | 16.59-56.09-27.31 | 1056.95 | 64.03 | 0.673 |
| 4Group （1 2 1 2 ） | 86.74-83.38-84.47-98.51 | 34.7-4.3-12.9-7689.1 | 13.97-57.06-28.23-0.74 | 15.88-53.68-29.58-0.85 | 1087.17 | 30.22 | 0.739 |
| 5Group （1 2 1 1 2 ） | 82.64-73.86-71.94-80.67-99.21 | 42.1-6.0-3.8-20.7-14477.6 | 9.21-31.65-44.28-14.12-0.74 | 10.17-32.02-40.21-16.75-0.86 | 1088.47 | 1.30 | 0.646 |
| 6Group （0 1 2 1 0 2 ） | 77.99-80.18-77.92-83.54-58.07-98.92 | 765.0-22.2-3.6-14.0-17.5-10568.7 | 0.45-15.30-55.27-25.71-2.53-0.74 | 0.46-15.41-49.29-26.66-7.32-0.86 | 1085.99 | -2.48 | 0.712 |
| AIC（Akaike information criterion）， BIC^#2^（Bayesian Information Criterion）, E_k_（Relative entropy）， Avepp （Average posterior probability）。A good model fit is characterized by: (1) the correct classification advantage (OCC) for each class being greater than 5, and (2) a strong agreement between the posterior probability of group membership (*P_j_)* and the actual group membership probability (*π_j_*). | | | | | | | |

**TableS3 Evaluation indicators for the fitting effect of different aging trajectory models**

| Group | Class | *OCC* | *Avepp（%）* | *P_j_（%）* | *π_j_（%）* |
| --- | --- | --- | --- | --- | --- |
| 1Group | Class1 |  | 100.00 | 100.00 | 100.00 |
| 2Group | Class1 | 9.5 | 87.24 | 41.01 | 41.95 |
|  | Class2 | 6.2 | 89.53 | 58.99 | 58.05 |
| 3Group | Class1 | 30.2 | 85.72 | 14.86 | 16.59 |
|  | Class2 | 4.2 | 84.17 | 59.14 | 56.09 |
|  | Class3 | 13.7 | 83.78 | 26.00 | 27.31 |
| 4Group | Class1 | 34.7 | 86.74 | 13.97 | 15.88 |
|  | Class2 | 4.3 | 83.38 | 57.06 | 53.68 |
|  | Class3 | 12.9 | 84.47 | 28.23 | 29.58 |
|  | Class4 | 7689.1 | 98.51 | 0.74 | 0.85 |
| 5Group | Class1 | 42.1 | 82.64 | 9.21 | 10.17 |
|  | Class2 | 6.0 | 73.86 | 31.65 | 32.02 |
|  | Class3 | 3.8 | 71.94 | 44.28 | 40.21 |
|  | Class4 | 20.7 | 80.67 | 14.12 | 16.75 |
|  | Class5 | 14477.6 | 99.21 | 0.74 | 0.86 |
| 6Group | Class1 | 765.0 | 77.99 | 0.45 | 0.46 |
|  | Class2 | 22.2 | 80.18 | 15.30 | 15.41 |
|  | Class3 | 3.6 | 77.92 | 55.27 | 49.29 |
|  | Class4 | 14.0 | 83.54 | 25.71 | 26.66 |
|  | Class5 | 17.5 | 58.07 | 2.53 | 7.32 |
|  | Class6 | 10568.7 | 98.92 | 0.74 | 0.86 |
| AIC（Akaike information criterion）， BIC^#2^（Bayesian Information Criterion）, E_k_（Relative entropy）， Avepp （Average posterior probability）。  Good model fit is characterized by: (1) the odds of correct classification (OCC) for each class being greater than 5, (2) a good consistency between the posterior probability of group membership (*P_j_*) and the actual probability of group membership (*π_j_*). | | | | | |

**TableS4 Aging trajectory model parameter estimation**

| Group | Class | Parameter | *β* | *SE* | *t* | *P* |
| --- | --- | --- | --- | --- | --- | --- |
| 1Group | Class.1 | Intercept | -0.10570 | 0.00660 | -16.015 | 0.00000 |
|  |  | Linear | 0.05133 | 0.00859 | 5.976 | 0.00000 |
|  |  | Quadratic | -0.00381 | 0.00214 | -1.780 | 0.07513 |
| 2Group | Class.1 | Intercept | -0.22002 | 0.00995 | -22.113 | 0.00000 |
|  |  | Linear | 0.03426 | 0.00330 | 10.382 | 0.00000 |
|  | Class.2 | Intercept | -0.01420 | 0.00839 | -1.692 | 0.09068 |
|  |  | Linear | 0.03871 | 0.00276 | 14.025 | 0.00000 |
| 3Group | Class.1 | Intercept | -0.30307 | 0.01502 | -20.178 | 0.00000 |
|  |  | Linear | 0.03287 | 0.00501 | 6.561 | 0.00000 |
|  | Class.2 | Intercept | -0.12619 | 0.01023 | -12.335 | 0.00000 |
|  |  | Linear | 0.05731 | 0.00939 | 6.103 | 0.00000 |
|  |  | Quadratic | -0.00470 | 0.00234 | -2.009 | 0.04469 |
|  | Class.3 | Intercept | 0.05147 | 0.01362 | 3.779 | 0.00016 |
|  |  | Linear | 0.09706 | 0.03132 | 3.099 | 0.00196 |
|  |  | Quadratic | -0.03939 | 0.02023 | -1.947 | 0.00000 |
|  |  | Cubic | 0.00611 | 0.00333 | 1.835 | 0.00000 |
| 4Group | Class.1 | Intercept | -0.30801 | 0.01495 | -20.603 | 0.00000 |
|  |  | Linear | 0.03321 | 0.00500 | 6.642 | 0.00000 |
|  | Class.2 | Intercept | -0.13257 | 0.01013 | -13.087 | 0.00000 |
|  |  | Linear | 0.05823 | 0.00935 | 6.228 | 0.00000 |
|  |  | Quadratic | -0.00510 | 0.00234 | -2.179 | 0.02939 |
|  | Class.3 | Intercept | 0.04004 | 0.01156 | 3.464 | 0.00054 |
|  |  | Linear | 0.03995 | 0.00367 | 10.886 | 0.00000 |
|  | Class.4 | Intercept | 0.20460 | 0.05511 | 3.713 | 0.00021 |
|  |  | Linear | 0.34714 | 0.06489 | 5.350 | 0.00000 |
|  |  | Quadratic | -0.11229 | 0.01578 | -7.116 | 0.00000 |
| 5Group | Class.1 | Intercept | -0.34100 | 0.01968 | -17.327 | 0.00000 |
|  |  | Linear | 0.03384 | 0.00622 | 5.441 | 0.00000 |
|  | Class.2 | Intercept | -0.18854 | 0.02297 | -8.208 | 0.00000 |
|  |  | Linear | 0.06459 | 0.01301 | 4.965 | 0.00000 |
|  |  | Quadratic | -0.00782 | 0.00321 | -2.436 | 0.01491 |
|  | Class.3 | Intercept | -0.06268 | 0.02452 | -2.556 | 0.01064 |
|  |  | Linear | 0.04103 | 0.00365 | 11.241 | 0.00000 |
|  | Class.4 | Intercept | 0.07381 | 0.01707 | 4.324 | 0.00002 |
|  |  | Linear | 0.04032 | 0.00519 | 7.769 | 0.00000 |
|  | Class.5 | Intercept | 0.20428 | 0.05328 | 3.834 | 0.00013 |
|  |  | Linear | 0.34745 | 0.06326 | 5.492 | 0.00000 |
|  |  | Quadratic | -0.11245 | 0.01533 | -7.335 | 0.00000 |
| 6Group | Class.1 | Intercept | -0.48276 | 0.06464 | -7.468 | 0.00000 |
|  | Class.2 | Intercept | -0.30791 | 0.01775 | -17.347 | 0.00000 |
|  |  | Linear | 0.03759 | 0.00526 | 7.146 | 0.00000 |
|  | Class.3 | Intercept | -0.12723 | 0.01067 | -11.924 | 0.00000 |
|  |  | Linear | 0.06376 | 0.01018 | 6.263 | 0.00000 |
|  |  | Quadratic | -0.00521 | 0.00245 | -2.127 | 0.03356 |
|  | Class.4 | Intercept | 0.05031 | 0.01181 | 4.260 | 0.00002 |
|  |  | Linear | 0.03878 | 0.00381 | 10.178 | 0.00000 |
|  | Class.5 | Intercept | -0.12024 | 0.02387 | -5.037 | 0.00000 |
|  | Class.6 | Intercept | 0.20454 | 0.05355 | 3.820 | 0.00014 |
|  |  | Linear | 0.34722 | 0.06334 | 5.482 | 0.00000 |
|  |  | Quadratic | -0.11236 | 0.01538 | -7.306 | 0.00000 |

**TableS5 Evaluation of the fitting effect of each trajectory group in the aging trajectory model**

| Group | Class | Curve equation |
| --- | --- | --- |
| 1 Group | Class1 | ŷ= 0.00004-0.00004x+0.00001x^2^ |
| 2 Group | Class1 | ŷ= 0.00010-0.00002x |
| 2 Group | Class2 | ŷ= 0.00003+0.00000x |
| 3 Group | Class1 | ŷ= 0.00023-0.00005x |
| 3 Group | Class2 | ŷ= 0.00005-0.00000x+0.00000x^2^ |
| 3 Group | Class3 | ŷ= 0.00004+0.00003x-0.00002x^2^+0.00000x^3^ |
| 4 Group | Class1 | ŷ= 0.00022-0.00005x |
| 4 Group | Class2 | ŷ= 0.00004-0.00000x+0.00000x^2^ |
| 4 Group | Class3 | ŷ= 0.00004-0.00000x |
| 4 Group | Class4 | ŷ= 0.00000+0.00000x-0.00000x^2^ |
| 5 Group | Class1 | ŷ= 0.00039-0.00006x |
| 5 Group | Class2 | ŷ= 0.00022+0.00000x+0.00000x^2^ |
| 5 Group | Class3 | ŷ= 0.00020-0.00000x |
| 5 Group | Class4 | ŷ= 0.00008+0.00001x |
| 5 Group | Class5 | ŷ= -0.00000-0.00000x+0.00000x^2^ |
| 6 Group | Class1 | ŷ= 0.00418 |
| 6 Group | Class2 | ŷ= 0.00039-0.00001x |
| 6 Group | Class3 | ŷ= 0.00007-0.00000x+0.00000x^2^ |
| 6 Group | Class4 | ŷ= 0.00004-0.00000x |
| 6 Group | Class5 | ŷ= 0.00021 |
| 6 Group | Class6 | ŷ= -0.00000+0.00000x-0.00000x^2^ |
|  | | |

**TableS6 Curve equations of each trajectory group within the aging trajectory model**


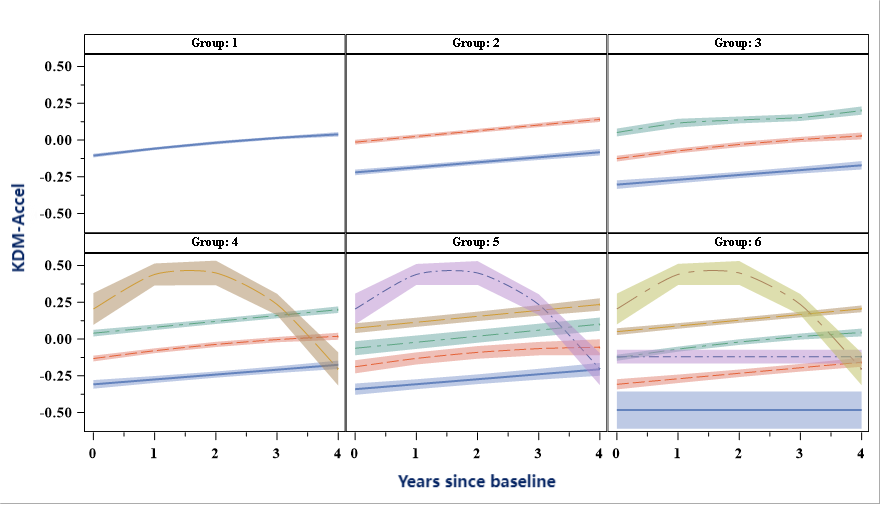


**Fig.S3.Figure of each group's aging trajectory fitting**


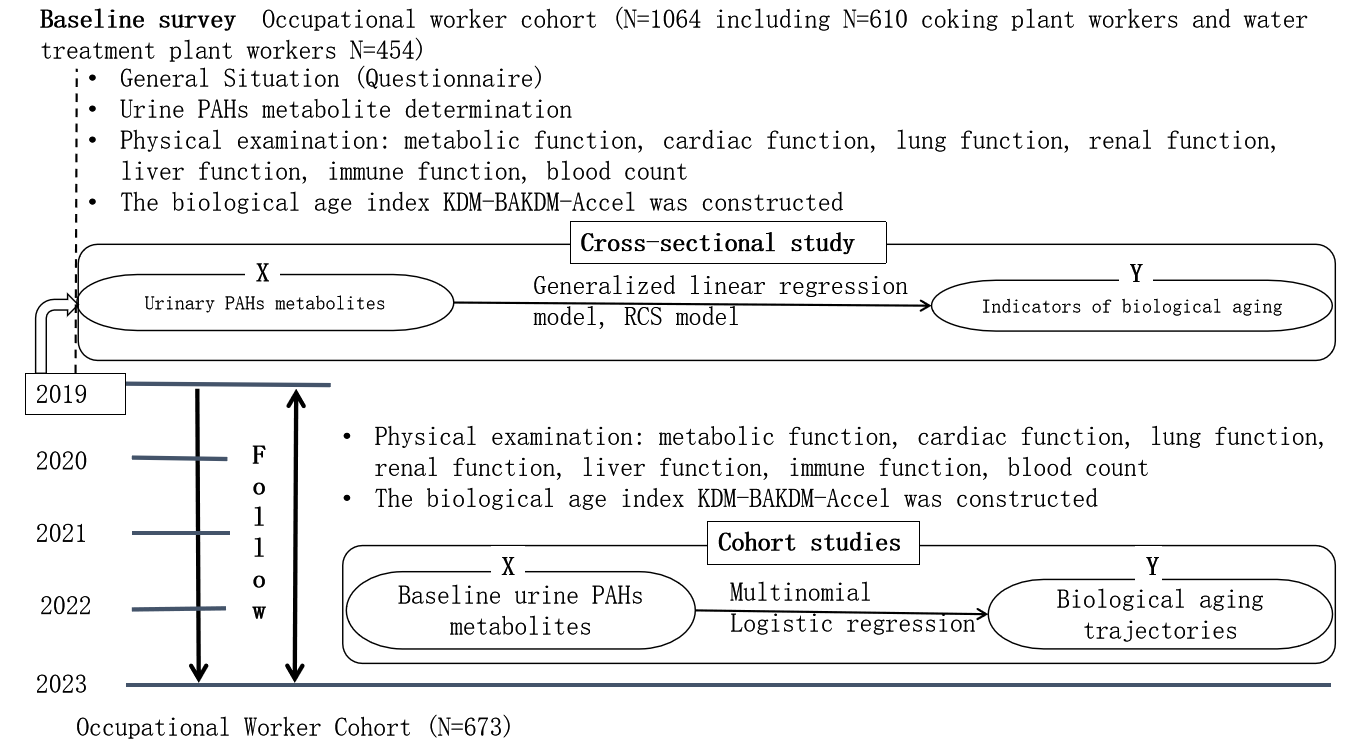


**Fig.S4. Technology Roadmap**

| variable | Total population | Coke Plant | Water Treatment Plant | *P* |
| --- | --- | --- | --- | --- |
|  | n=673 | n=383 | n=290 |  |
| Age (years) | 44.71 ± 7.32 | 43.07 ± 7.96 | 46.89 ± 5.71 | **<0.001** |
| gender |  |  |  | **0.017** |
| man | 602（89.45） | 352（91.91） | 250（86.21） |  |
| woman | 71（10.55） | 31（8.09） | 40（13.79） |  |
| Educational attainment (years, N%) |  |  |  | 0.401 |
| 1-9 | 176（26.15） | 95（24.80） | 81（27.93） |  |
| 10-12 | 421（62.56） | 240（62.66） | 181（62.41） |  |
| ≥13 | 76（11.29） | 48（12.53） | 28（9.66） |  |
| marital status  (Yes, N%) |  |  |  | 0.315 |
| Yes | 636（94.50） | 359（93.73） | 277（95.52） |  |
| No | 37（5.50） | 24（6.27） | 13（4.48） |  |
| Smoking  (Yes, N%) | 409（60.77） | 171（44.65） | 148（51.03） | 0.100 |
| Alcohol consumption  (Yes, n%) | 275（40.86） | 161（42.04） | 114（39.31） | 0.476 |
| Tea drinking condition  (Yes, n%) | 426（63.30） | 232（60.57） | 194（66.90） | 0.092 |
| Exercise status |  |  |  | **<0.001** |
| never | 186（27.64） | 126（32.90） | 60（20.69） |  |
| Occasionally | 243（36.11） | 132（34.46） | 111（38.28） |  |
| often | 244（36.26） | 125（32.64） | 119（41.03） |  |
| Night shift (yes, n%) | 467（69.60） | 272（71.02） | 195（67.71） | 0.356 |
| KDM (years) |  |  |  |  |
| kdm_advance (years old) | 0.68 ± 1.22 | 0.94 ± 1.49 | 0.35 ± 0.53 | **<0.001** |
| Urine PAHs metabolites (μg/L) | 0.25 ± 1.16 | 0.36 ± 1.53 | 0.11 ± 1.14 | **0.003** |
| 2-OHNap | 0.09 ± 0.15 | 0.12 ± 0.18 | 0.07 ± 0.08 | **<0.001** |
| 1-OHNap | 0.15 ± 0.18 | 0.18 ± 0.20 | 0.12 ± 0.15 | **<0.001** |
| 3-OHFlu | 0.28 ± 0.47 | 0.40 ± 0.59 | 0.11 ± 0.09 | **<0.001** |
| 2 OHFlu | 0.42 ± 0.55 | 0.46 ± 0.67 | 0.37 ± 0.35 | 0.173 |
| 2-OHPhe | 0.10 ± 0.16 | 0.12 ± 0.18 | 0.08 ± 0.13 | **<0.001** |
| 9-OHPhe | 0.20 ± 0.25 | 0.24 ± 0.32 | 0.15 ± 0.09 | 0.161 |
| 1-OHPhe | 0.06 ± 0.09 | 0.07 ± 0.12 | 0.04 ± 0.03 | 0.833 |
| 1-OHPyr | 0.08 ± 0.08 | 0.09 ± 0.09 | 0.08 ± 0.06 | **0.017** |
| 3-OHChr | 0.06 ± 0.09 | 0.07 ± 0.10 | 0.05 ± 0.06 | 0.221 |
| 6-OHChr | 2.19 ± 2.67 | 2.82 ± 3.28 | 1.37 ± 1.04 | **<0.001** |
| 9-OHBap | 44.61 ± 7.33 | 42.97 ± 7.97 | 46.77 ± 5.74 | **<0.001** |
| Ʃ-OH PAHs | -0.11 ± 0.17 | -0.09 ± 0.18 | -0.12 ± 0.16 | 0.321 |
| Note: Bold represents *P*<0.05;  The continuous data were expressed as x±s, and the statistical differences were compared by ANOVA.  The numerical data were expressed as n (%), and the chi-square test was used to compare the statistical differences. | | | | |

**TableS7 Basic Characteristics of Coke Plant Queue Workers(n=673)**

| Urine metabolites of OH-PAHs | Moderate aging vs. slow aging |  |  | High aging vs. slow aging |  |
| --- | --- | --- | --- | --- | --- |
|  | OR （95% CI） | *P* |  | OR （95% CI） | *P* |
| Ʃ-OHPAHs ^a^ | **1.453（1.001,2.108）** | **0.049** |  | **1.631（1.082,2.459）** | **0.019** |
| 2-OHNAP ^b^ | 1.185（0.937,1.497） | 0.156 |  | 0.981（0.771,1.247） | 0.875 |
| 1-OHNAP ^b^ | 0.893（0.642,1.242） | 0.502 |  | 0.985（0.688,1.408） | 0.932 |
| 3-OHFLU ^b^ | 1.142（0.867,1.505） | 0.344 |  | 1.158（0.864,1.551） | 0.328 |
| 2-OHFLU ^b^ | **0.622（0.390,0.991）** | **0.046** |  | **0.565（0.337,0.946）** | **0.030** |
| 2-OHPHE ^b^ | 1.118（0.878,1.423） | 0.366 |  | **1.622（1.156,2.277）** | **0.005** |
| 9-OHPHE ^b^ | 1.255（0.807,1.950） | 0.313 |  | 1.267（0.785,2.045） | 0.332 |
| 1-OHPHE ^b^ | 0.999（0.760,1.312） | 0.993 |  | 0.868（0.614,1.227） | 0.422 |
| 1-OHPYR ^b^ | **1.467（1.010,2.131）** | **0.044** |  | **1.503（1.113,2.029）** | **0.008** |
| 3-OHChr ^b^ | 0.759（0.536,1.076 ） | 0.122 |  | 0.829（0.545,1.262） | 0.383 |
| 6-OHChr ^b^ | 1.125（0.862,1.468） | 0.388 |  | 1.015（0.707,1.457） | 0.937 |
| 9-OHBap ^b^ | 1.050（0.830,1.328） | 0.685 |  | 1.208（0.892,1.636） | 0.222 |

**Table S8 The Relationship Between Urinary OH-PAHs Metabolites and the Aging Trajectory in Male Occupational Workers**

Slow aging: Slow aging trajectory, Moderate aging: Moderately accelerated aging trajectory, High aging: Highly accelerated aging trajectory.

Model: a Adjusted for age, education level, smoking status, alcohol consumption, tea consumption, physical activity, monthly income, and night shift status. b Adjusted for age, education level, smoking status, alcohol consumption, tea consumption, physical activity, monthly income, night shift status, and other urinary OH-PAH metabolites. *P*<0.05.

| Urine metabolites of OH-PAHs | Moderate aging vs. slow aging |  |  | High aging vs. slow aging |  |
| --- | --- | --- | --- | --- | --- |
|  | OR （95% CI） | *P* |  | OR （95% CI） | *P* |
| Ʃ-OHPAHs ^a^ | 1.097（0.470,2.561） | 0.830 |  | 1.333（0.209,8.485） | 0.761 |
| 2-OHNAP ^b^ | 0.854（0.408,1.789） | 0.676 |  | 0.659（0.156,2.783） | 0.570 |
| 1-OHNAP ^b^ | 1.006（0.342,2.964） | 0.991 |  | 1.218（0.421,3.525） | 0.716 |
| 3-OHFLU ^b^ | 2.597（0.880,7.661） | 0.084 |  | 1.457（0.615,3.450） | 0.392 |
| 2-OHFLU ^b^ | **0.226（0.056,0.909）** | **0.036** |  | **0.044（0.002,0.904）** | **0.043** |
| 2-OHPHE ^b^ | 1.314（0.593,2.907） | 0.501 |  | 0.725（0.231,2.277） | 0.581 |
| 9-OHPHE ^b^ | 0.569（0.089,3.647） | 0.552 |  | 0.158（0.008,3.049） | 0.222 |
| 1-OHPHE ^b^ | 1.660（0.480,5.741） | 0.424 |  | 0.436（0.157,1.209） | 0.111 |
| 1-OHPYR ^b^ | 2.520（0.592,10.727） | 0.211 |  | 3.113（0.7,13.832） | 0.136 |
| 3-OHChr ^b^ | 0.660（0.126,3.446 ） | 0.622 |  | 1.716（0.794,3.709） | 0.170 |
| 6-OHChr ^b^ | 2.150（0.660,6.999） | 0.204 |  | 1.966（0.322,12.011） | 0.464 |
| 9-OHBap ^b^ | 0.759（0.325,1.773 ） | 0.524 |  | 4.145（0.629,27.307） | 0.139 |

**Table S9 The Relationship Between Urinary OH-PAHs Metabolites and the Aging Trajectory in Female Occupational Workers**

Slow aging: Slow aging trajectory, Moderate aging: Moderately accelerated aging trajectory, High aging: Highly accelerated aging trajectory.

Model: a Adjusted for age, education level, smoking status, alcohol consumption, tea consumption, physical activity, monthly income, and night shift status. b Adjusted for age, education level, smoking status, alcohol consumption, tea consumption, physical activity, monthly income, night shift status, and other urinary OH-PAH metabolites. *P*<0.05.
